# Children’s and Adolescent’s Use of Context in Judgments of Emotion Intensity

**DOI:** 10.1007/s42761-024-00279-5

**Published:** 2024-09-26

**Authors:** Brian T. Leitzke, Aaron Cochrane, Andrea G. Stein, Gwyneth A. DeLap, C. Shawn Green, Seth D. Pollak

**Affiliations:** 1 Department of Psychology, University of WI–Madison, Waisman Center, 1500 Highland Avenue, Room 399, Madison, WI 53705 USA; 2 Department of Cognitive and Psychological Sciences, Brown University, 190 Thayer St, Providence, RI 02912 USA; 3 University of Rochester, 494 Meliora Hall, Rochester, NY 14627 USA

**Keywords:** Emotion recognition, Emotion labeling, Emotion intensity, Development, Context

## Abstract

The ability to infer others’ emotions is important for social communication. This study examines three key aspects of emotion perception for which relatively little is currently known: (1) the evaluation of the intensity of portrayed emotion, (2) the role of contextual information in the perception of facial configurations, and (3) developmental differences in how children perceive co-occurring facial and contextual information. Two experiments examined developmental effects on the influence of congruent, incongruent, and neutral situational contexts on participants’ reasoning about others’ emotions, both with and without emotion labels. Experiment 1 revealed that participants interpreted others’ emotions to be of higher intensity when facial movements were congruent with contextual information. This effect was greater for children compared to adolescents and adults. Experiment 2 showed that without verbal emotion category labels, adults relied less on context to scale their intensity judgments, but children showed an opposite pattern; in the absence of labels, children relied more on contextual information than facial information. Making accurate inferences about others’ internal states is a complex learning task given high variability within and across individuals and contexts. These data suggest changes in attention to perceptual information as such learning occurs.

**Supplementary Information:**

The online version contains supplementary material available at 10.1007/s42761-024-00279-5.

To make accurate inferences about another person’s emotions, children must learn to construct meaning by integrating myriad signals. These signals include facial musculature, bodily movements, situational contexts, vocal cues, and memories and experiences of past events (Ruba & Pollak, 2020; Sauter et al., 2010; Van den Stock & de Gelder, 2012). Most research on emotion perception focuses on peak prototypes of facial movements with little attention to emotion intensity. Yet full-blown facial displays are not often encountered in the real world (Calvo & Nummenmaa, 2016). Rather, real-world emotion perception requires more nuanced judgments, such as whether someone might be merely annoyed, increasingly irritated, or enraged (Calder et al., 2000). Adding to this complexity, facial movements must be interpreted within the situational contexts in which they are embedded (Aviezer et al., 2012; Mesquita, 2022; Martinez, 2019), and this interpretation is dependent on experience (Pollak et al., 2009). Here, we examine age-related differences in the effect of context, in terms of scenes of situations, on individuals’ interpretations of others’ emotional intensity.

To accurately predict someone else’s behavior, one must often gauge the intensity of the other person’s emotions. While we often focus on facial configurations to make these assessments, facial muscular activation exists on continua that afford meaningful distinctions beyond categorical labeling (Martinez, 2017), and even with precise attention to facial movements, interpretive judgments about emotion are dependent upon social context (Barrett et al., 2019; Chen & Whitney, 2019). Such contextual influence on face perception has been found in both laboratory and observational studies and has been reported across development (Aviezer et al., 2012; Noh & Isaacowitz, 2013; Rajhans et al., 2016; Reschkle & Walle, 2021). While older children and adults tended to allocate the majority of their attention toward faces relative to contextual information, younger children divide their attention to both sources of information equally, suggesting a developmental trend of increasing prioritization of facial over contextual information with age (Leitzke & Pollak, 2016). Yet contextual influences on perceptions of intensity remain less understood.

## Context and Ratings of Emotion Intensity

Studies examining the effects of context on emotion judgments predominantly rely on emotion categorization paradigms. High-intensity faces are more accurately categorized than low-intensity faces (Calder et al., 2000; Gao & Maurer, 2009; Hess et al., 1997). Yet, context plays an influential role in how these stimuli are categorized (Aviezer et al., 2012), and may play a role in perceptions of emotional intensity as well. Conceptually, this makes sense: perceivers may use situational contexts to gauge how strongly they think an emoter ought to feel given the circumstances. We are likely to respond differently if we think another person is startled versus terrified, or if we interpret them as disappointed versus devastated. However, the extent to which context influences ratings of emotion intensity has yet to be directly examined.

## Current Study

There is little research examining age-related differences in the effect of context on intensity ratings of perceived emotion. In two experiments, we tested children, adolescents, and young adults as they viewed facial configurations presented with emotion-congruent, incongruent, and neutral contexts. Based upon extant literature, we predicted that ratings of intensity would be greater when faces were presented in a congruent, relative to an incongruent or neutral context and that this effect would be more prominent in younger, compared to older, participants. In the second experiment, we replicated the first experiment without providing participants with any emotion labels. Based upon the findings from Experiment 1, we expected to replicate the intensity-congruency effect but also predicted that the absence of labels would further increase children’s reliance on contextual information.

## Experiment 1: Does Congruency Between Faces and Contexts Influence the Intensity of Perceived Emotion?

### Method

#### Participants

One hundred sixty-two individuals from three age groups were recruited for this study. Children (*N* = 56; *M*_age_ = 7 years, 11 months, *SD* = 7 months; range 7.02–9.12; 48% Female; 7% Asian or Asian-American, 6% Black or African-American, 7% Hispanic, 73% White, and 7% Other Racial/Ethnic identification) and pre-adolescents (*N* = 54; *M*_age_ = 13 years, 1 month, *SD* = 7 months; range 11.86–13.93; 44% Female; 8% Asian or Asian-American, 6% Black or African-American, 4% Hispanic, 81% White, and 1% Other Racial/Ethnic identification) were recruited from the local community via television commercials, radio ads, and posted flyers as well as through a local school district registry where parents volunteered their contact information for research study recruitment. These ages were selected to be consistent with those used in Leitzke and Pollak (2016). Young adults (*N* = 52; *M*_age_ = 19 years, 7 months, *SD* = 11 months; range 17.97–21.99; 37% Female; 26% Asian or Asian-American, 6% Black or African-American, 8% Hispanic, 58% White, and 2% Other Racial/Ethnic identification) were recruited from an introductory psychology course or from the campus community via posted flyers. Power analyses are reported below in the Data Analytic Plan.

#### Stimuli

##### Facial stimuli

**Fig. 1:**
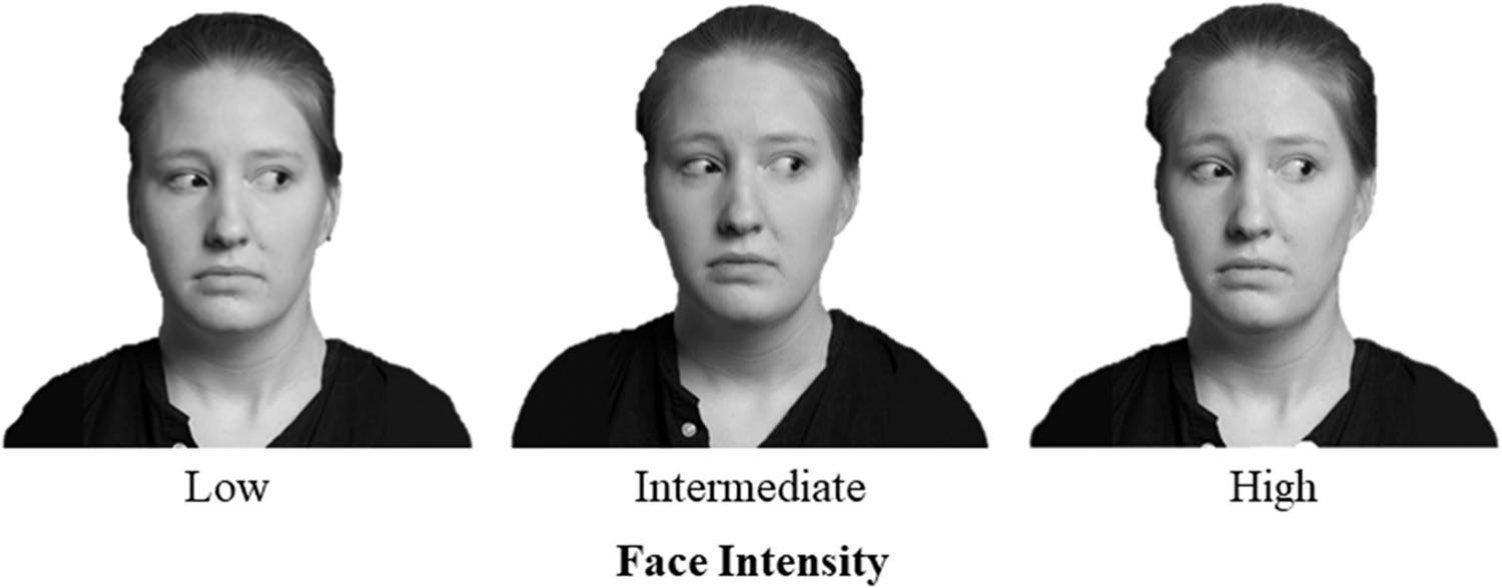
Example of low, intermediate, and high intensity for facial configurations associated with fear

Facial stimuli were borrowed from the Interdisciplinary Affective Science Laboratory (IASLab) Facial Stimuli Set.^1^ We included models with averted gaze to direct participants’ attention toward the contextual information displayed in each image. We selected prototypical categories of anger, disgust, fear, and sadness from four models (two females: models F17 and F19, and two males: models M01 and M07). This stimulus set did not require models to self-identify their race, though a pilot study demonstrated that all selected models were unanimously identified as White in a free-response format. We selected all White models to reduce biases that may be due to race (Elfenbien & Ambady, 2002). While biases by gender also exist (Adams et al., 2015), we included both male and female models to ensure some extent of natural variability in the presented stimulus set. To create variation in emotion intensity, we morphed the facial displays of each model with that same model’s neutral expression. We used j.psychomorph (see Tiddeman et al., 2005) to morph each image to create 10% increments in intensity and selected the 20%, 50%, and 80% images to represent, low-, intermediate-, and high-intensity stimuli respectively (Fig. 1).

##### Contextual stimuli

One hundred contextual images were downloaded from the Internet and rated by 301 adult participants via Mechanical Turk (see Buhrmester et al., 2011). Participants viewed each image and assigned emotion labels to each one, choosing from 27 highly used emotion terms from different cultures (Srinivasan & Martinez, 2018). To avoid a forced-choice bias, participants were able to assign as many options as they felt were appropriate in response to how they believed someone might feel if they viewed each scenario in real life. The top-rated images for each emotion category were selected (all images achieved 50% endorsements for each emotion category; chance performance would be 3.7%). We selected the four top-rated images for each emotion prototypical category (anger, disgust, fear, happiness, sadness, neutral), for a total of 24 images. For each category, two contextual images included people and two did not include people. The faces of all people in the situational context were blurred.

##### Composites

**Fig. 2:**
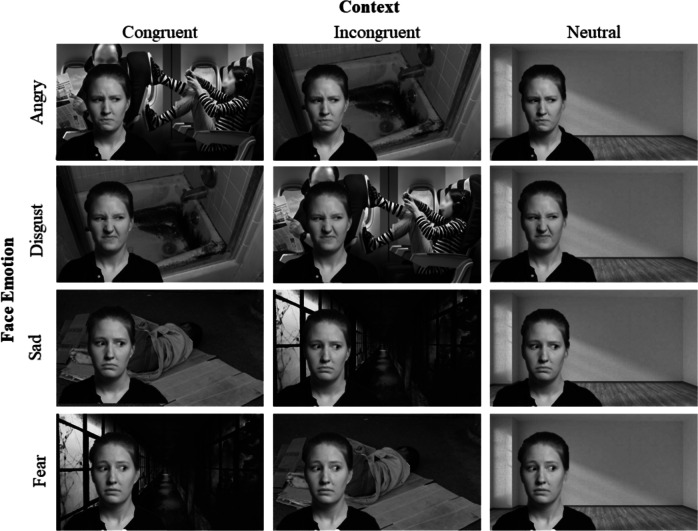
Exemplars of face emotion/context composites. All four emotions (anger, disgust, sadness, fear) are depicted in congruent, incongruent, and neutral contexts. The model depicted is model F17 from the International Affective Science Laboratory (IASLab) Face Set expressing high (80%) intensity for each facial configuration

Facial images were superimposed on top of each context to create composites of each face appearing to respond to a situation. Four facial categories (fear, sad, disgust, angry) were fully crossed with the three conditions (congruent face-context, incongruent face-context, neutral) resulting in 12 pairings. The congruent condition paired each face with a context of the same emotion (e.g., anger face within anger context). Incongruent pairings were chosen based on the confusability of anger and disgust (Aviezer et al., 2012) as well as sadness and fear (Mondloch, 2012); anger faces were paired with disgust contexts, and vice versa, and sad faces were paired with fear faces, and vice versa. All 12 face/context pairings were fully crossed with three intensities (low, intermediate, high), creating 36 face emotion/congruence/intensity composites (Fig. 2).

Each face emotion/congruence/intensity composite appeared evenly with the four different models (two males, two females) and context exemplars (two with people, two without). One-hundred forty-four trials comprised of four presentations of each of the 36-face emotion/congruence/intensity composites, each with a different model and context exemplar. Sixteen trials consisted of smiling faces and contexts to provide variation in stimuli valence. Specifically, smiling faces were morphed to create low (20%) and high (80%) intensity smiling faces in congruent and neutral contexts paired with each of the four facial emotion posers. In total, this experiment consisted of 160 total trials, with each of the four models and contexts appearing together ten times across the experiment.

#### Judgment Task

Participants viewed each image for 1,000 ms before being asked to rate the image. Participants responded to the question “How is this person feeling?” by using a computer mouse to move a cursor along a visual analog scale that ranged from “not at all (displayed facial emotion)?” to “extremely (displayed facial emotion)?” to indicate the intensity of emotion they believed each person was experiencing. Participants were randomly assigned to rate increasing intensity from left to right or right to left to reduce any directional bias. The resulting scores ranged from 0 (not at all displaying that emotion) to 100 (displaying that emotion very intensely). Participants were instructed to focus explicitly on the face in each image when making their ratings to ensure ratings reflected the intensity of emotion in different contexts. The experiment was divided into four 40-trial blocks, which participants viewed in a random order to ensure that all participants saw all stimuli and to control for order effects. At the end of the task, participants also completed an additional block consisting of 48 trials of all face emotion/intensity/model combinations presented in the absence of any contextual information. The task was created and presented with E-Prime 3.0 software (Psychology Software Tools, Pittsburgh, PA).

#### Procedure

The University of Wisconsin–Madison Institutional Review Board approved all procedures, and all participants provided informed written consent/assent. Participants and parents completed (as appropriate for the participants’ ages) consent/assent procedures in a waiting room before moving to an individual testing room where they completed the study tasks. Participants completed all four blocks of the judgment task before completing the no-context block. Stimuli were presented on a 21-in. Dell computer monitor at a resolution of 1920 by 1080 pixels and displayed at 75% of the width and height of the screen. A research assistant remained with each participant throughout the study to encourage and remind them to pay attention to the change in emotion label and direction of the scale and to use the face of each individual to make their determination of emotion intensity. Adult participants were compensated with either course credit, if recruited from the psychology course, or a $10 cash payment if recruited from the campus community. Children received a prize for completing this experiment, and their parents were compensated with a $20 cash payment.

#### Data Analytic Plan

We excluded trials with response times under 200 ms, as these would have occurred prior to the time required to initiate perceptual and motor processes following stimulus presentation (Whelan, 2008; 0.1% of all responses). We also excluded all trials involving the two contexts depicting anger in a non-social setting, based on results from a post hoc validation study conducted with children (*n* = 129) and adolescents (*n* = 51) on Lookit (Scott & Schulz, 2017). This study showed that while the stimuli were effective for adults, children did not consistently perceive these two contexts as anger-inducing; less than 50% of children endorsed the expected label (“mad”) for these stimuli (ratings available online on OSF).

Data were analyzed in R (R Core Team, 2018) using the brms package (Bürkner, 2017) to implement a Bayesian fitting approach. To do so, we fit a Bayesian hierarchical beta regression with monotonic coefficients (model code available online on OSF). Beta regression, in which fit values may range from zero to one, includes a dispersion parameter *phi* which we modeled as a by-age-group random effect. Additional random intercepts were included for ratings by participant and by context emotion. Random intercepts and linear emotion-intensity slopes were estimated for each combination of face model and emotion. Default priors were used in all cases. No aggregation was completed prior to data fitting (i.e., models were fit to raw by-trial data). Monotonic coefficients are used to estimate the overall fixed effects of an ordered (monotonic) variable when the relative distance between the levels is unknown (Bürkner & Charpentier, 2020). The overall effect was estimated and reported here and represents half of the difference between low-intensity and high-intensity faces or between incongruent and congruent contexts. The monotonic regression allowed the overall effect to account for a set of simplex parameters which controlled for variations in distances between levels (e.g., low-intensity, medium-intensity, or high-intensity). Monotonic coefficients were estimated for face intensity as well as context congruency.

Models were fit using two chains run for 5,000 iterations each and discarding the first 2500 samples as warmup. From these fits, we extracted values for specific groups and trial types for each sample of parameters, corresponding to 5,000 separate estimates of these values. We then tested for differences in ratings by examining the overlaps in the distributions of parameter samples (Kruschke, 2013). By subtracting values in paired samples, we calculated distributions corresponding to predicted differences between two groups (e.g., children vs. adults) or trial types (e.g., low vs. high intensity). If the distribution of differences was largely positive or negative, we considered the difference reliable (using conventional two-tailed 95% quantiles of parameter differences). We reported these comparisons in terms of the median, as mean values are subject to bias with potentially skewed distributions, and in terms of 95% credible intervals of the differences. We also calculated the proportion of posterior parameter samples that were on the opposite side of zero as the median parameter value (i.e., evidence for an effect in the opposite direction then the median). Finally, we multiplied this proportion of samples by two to be on the same scale as typical frequentist *p*-values and report the value as *Bayesian-p*.

To calculate our power to detect fixed effects, we used a maximum-likelihood simulation-based approach (Green & MacLeod, 2016). While our primary models include monotonic effects (e.g., of congruency), to reduce the computational burden, we used linear effects of congruency and emotion intensity. For the simulation-based power analyses, we fit a generalized linear mixed-effect model to the original data. Then, using that fit to the real data, one fixed-effect point estimate at a time was replaced with a coefficient of a prespecified size (e.g., the interaction between face intensity and background congruency was set to be 0.05 in one simulation). From this model in which one coefficient was specified and many aspects had been empirically estimated (e.g., error variance; random-effects estimates), data was repeatedly simulated, and the same model fit. This allowed for an estimate of the probability of null hypothesis rejection for that coefficient at that magnitude. By repeating the process for various magnitudes and for different coefficients, we estimated the size of the fixed-effect coefficients at which we would have 80% power to detect a true effect. The results of power analyses are reported in Supplemental Table 3.

### Results

We first excluded all participants who had an average rating for low-intensity faces that was higher than their average rating for high-intensity faces, which we interpreted as non-compliance with task instructions (remaining participants: children: *n* = 40, adolescents: *n* = 54, adult: *n* = 52). The two model chains, each run for 5,000 iterations, demonstrated convergent parameter distributions (max fixed-effect r-hat < 1.01). Summary statistics are reported in Table 1.

**Table 1:** Summary statistics for model-fitted intensity ratings in congruent, incongruent, and neutral contexts by face intensity and age group in experiment 1

				95% CI	
Congruence	Intensity	Age Group	Median	Lower	Upper
Congruent	Low	Adolescents	0.438	0.241	0.615
		Adult	0.482	0.283	0.659
		Children	0.587	0.374	0.748
	Intermediate	Adolescents	0.675	0.484	0.802
		Adult	0.687	0.493	0.811
		Children	0.744	0.573	0.849
	High	Adolescents	0.759	0.567	0.871
		Adult	0.757	0.567	0.868
		Children	0.77	0.586	0.875
Neutral	Low	Adolescents	0.395	0.207	0.577
		Adult	0.436	0.247	0.622
		Children	0.459	0.26	0.641
	Intermediate	Adolescents	0.641	0.444	0.779
		Adult	0.646	0.45	0.781
		Children	0.654	0.46	0.789
	High	Adolescents	0.735	0.535	0.854
		Adult	0.725	0.523	0.847
		Children	0.693	0.484	0.827
Incongruent	Low	Adolescents	0.375	0.197	0.551
		Adult	0.406	0.225	0.595
		Children	0.434	0.241	0.616
	Intermediate	Adolescents	0.62	0.422	0.76
		Adult	0.615	0.416	0.759
		Children	0.684	0.498	0.809
	High	Adolescents	0.72	0.517	0.844
		Adult	0.698	0.495	0.83
		Children	0.733	0.538	0.854

Values represent log odds. Intensity levels represent 20% (low), 50% (intermediate), and 80% (high) morphs

#### Hypothesis #1: Contextual Congruency Will Result in More Intense Perceived Emotion

**Fig. 3:**
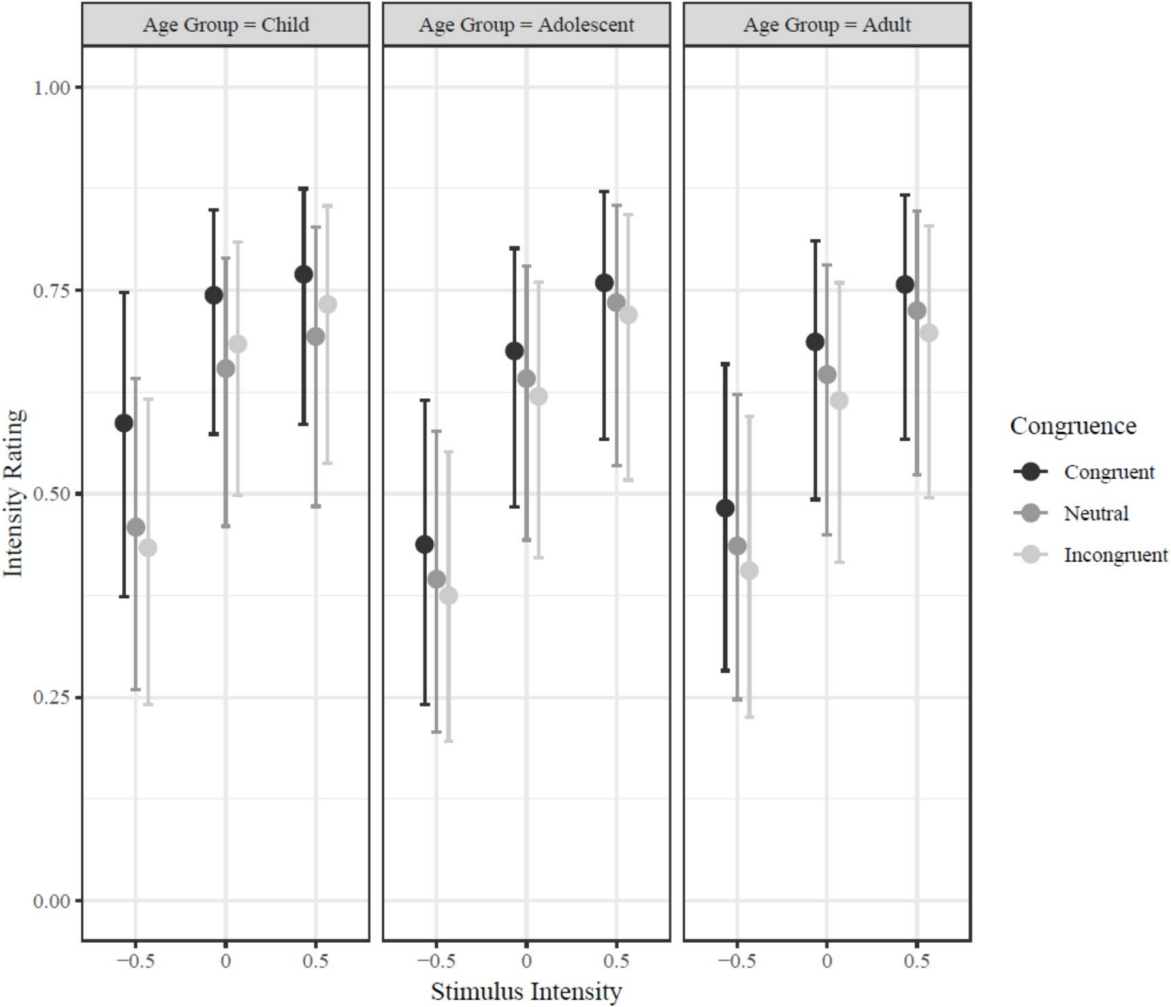
Predicted intensity ratings in Experiment 1 for congruent, neutral, and non-congruent contexts by face intensity (− 0.5 = low, 0 = intermediate, 0.5 = high) for adolescents, adults, and children with emotion labels provided. Predicted ratings reflect posterior samples calculated from generalized, multi-level Bayesian regression. Error bars represented 95% credible intervals (meaning that for each interval, there is a 95% probability the true mean intensity rating for the corresponding age group and stimulus intensity level falls within the depicted range). Note that error bars cannot be used to determine reliability because they reflect within- and between-subject variation, whereas reliability of fixed-effect coefficients controlled for between-subject variation

Across all age groups, faces in congruent contexts were rated as conveying more intense feelings than faces presented with incongruent contexts (md = 0.249; CI_95_ = [0.200, 0.294], *Bayesian-p* < .001). This was true for all three age groups (children: md = 0.309; CI_95_ = [0.252, 0.364], *Bayesian-p* < .001; adolescents: md = 0.129; CI_95_ = [0.076, 0.174], *Bayesian-p* < .001; adults: md = 0.155; CI_95_ = [0.096, 0.212], *Bayesian-p* < .001). When we examined ratings across levels of facial intensity, we found that this contextual congruency effect was stronger for low-intensity faces than for high-intensity faces (md = 0.095; CI_95_ = [0.056, 0.141], *Bayesian-p* < .001; see Fig. 3).

#### Hypothesis #2: Younger Children’s Perceived Intensity of Emotions Will Be More Influenced by Context Than Adolescents or Adults

As noted above, all three age groups rated faces as more intense in congruent contexts than in incongruent contexts. However, children demonstrated a stronger contextual congruency effect than adolescents (md = 0.180; CI_95_ = [0.123, 0.242], *Bayesian-p* < .001) or adults (md = 0.154; CI_95_ = [0.072, 0.234], *Bayesian-p* < .001). Adolescents and adults, on the other hand, showed comparable congruency-related differences in intensity ratings (md = 0.026; CI_95_ = [− 0.051, 0.095], *Bayesian-p* = .472; see Fig. 3).

### Summary of Experiment 1

Participants interpreted others’ emotions to be of higher intensity when facial movements were congruent with contextual information. This effect was greater for children compared to adolescents and adults, suggesting that context exerts an especially strong influence on the way children perceive emotions.

## Experiment 2: Does the Presence of Verbal Labels Influence the Intensity of Perceived Emotion?

Much research activity in affective science concerns the role of language and labeling in emotion reasoning. In Experiment 1, participants’ intensity ratings were anchored to a specific emotion label, as they rated intensity on a scale that ranged from “not at all angry/disgusted/sad/scared” to “extremely angry/disgusted/sad/scared.” Thus, participants’ intensity ratings were influenced by the categorical labels we supplied, even if the participant did not feel the individual in the image was actually experiencing that emotion. To inform our understanding of the impact of emotion labels on this task, Experiment 2 was conducted without the use of any specific emotion words. At the completion of the task, we asked participants to provide their own labels for the stimuli to get a sense of how similarly participants construed the stimuli (these analyses were not part of our a priori hypotheses but are reported in the Supplemental Material, see Supplementary Tables 1 and 2).

### Method

All stimuli, measures, and procedures were identical to Experiment 1 except that no emotion labels were provided in the task, as described in the “Emotion Judgment Task” section.

#### Participants

One hundred sixty-four individuals from three age groups were recruited for this study. Children (*N* = 55; *M*_age_ = 7 years, 10 months, *SD* = 7 months; 31% Female; 4% Asian or Asian-American, 11% Black or African-American, 9% Hispanic, 73% White, and 4% Other Racial/Ethnic identification) and pre-adolescents (*N* = 56; *M*_age_ = 12 years, 10 months, *SD* = 6 months; 64% Female; 5% Asian or Asian-American, 9% Black or African-American, 4% Hispanic, 80% White, and 2% Other Racial/Ethnic identification) were recruited from the local community via television commercials, radio ads, and posted flyers as well as through a local school district registry where parents volunteered their contact information for research study recruitment. Young adults (*N* = 53; *M*_age_ = 19 years, 6 months, *SD* = 11 months; 53% Female; 11% Asian or Asian-American, 11% Black or African-American, 13% Hispanic, and 64% White,) were recruited from an introductory psychology course or from the campus community via posted flyers.

#### Emotion Judgment Task

Participants viewed and rated images in the same manner as Experiment 1, however, they responded to the question “How much is this person feeling right now?” displayed above a visual analog scale ranging from “not at all” to “extremely.”

### Results

We processed and analyzed these data with the same procedures as in Experiment 1. As in Experiment 1, we excluded all participants who had an average response to low-intensity faces that was higher than their average rating of high-intensity faces (final *n*s: children: *n* = 48, adolescents: *n* = 52, adults: *n* = 49). The two model chains, each run for 5,000 iterations, demonstrated convergent parameter distributions (max fixed-effect r-hat = 1.006). Summary statistics are reported in Table 2.

**Table 2:** Summary statistics for model-fitted intensity ratings in congruent, incongruent, and neutral contexts by face intensity and age group in experiment 2

				95% CI	
Congruence	Intensity	Age Group	Median	Lower	Upper
Congruent	Low	Adolescents	0.428	0.245	0.608
		Adult	0.43	0.249	0.609
		Children	0.492	0.291	0.665
	Intermediate	Adolescents	0.601	0.421	0.751
		Adult	0.614	0.435	0.76
		Children	0.629	0.447	0.769
	High	Adolescents	0.654	0.468	0.805
		Adult	0.673	0.491	0.818
		Children	0.665	0.475	0.81
Neutral	Low	Adolescents	0.408	0.234	0.587
		Adult	0.422	0.245	0.602
		Children	0.476	0.283	0.652
	Intermediate	Adolescents	0.587	0.407	0.739
		Adult	0.583	0.408	0.733
		Children	0.591	0.407	0.738
	High	Adolescents	0.661	0.478	0.808
		Adult	0.657	0.472	0.807
		Children	0.631	0.441	0.789
Incongruent	Low	Adolescents	0.394	0.222	0.573
		Adult	0.422	0.243	0.602
		Children	0.52	0.316	0.691
	Intermediate	Adolescents	0.575	0.392	0.73
		Adult	0.566	0.389	0.72
		Children	0.627	0.445	0.766
	High	Adolescents	0.656	0.469	0.805
		Adult	0.643	0.458	0.797
		Children	0.666	0.481	0.812

Values represent log odds. Intensity levels represent 20% (low), 50% (intermediate), and 80% (high) morphs

#### Hypothesis #1: Context Congruency Will Result in More Intense Perceived Emotion, Even in the Absence of Emotion Labels

**Fig. 4:**
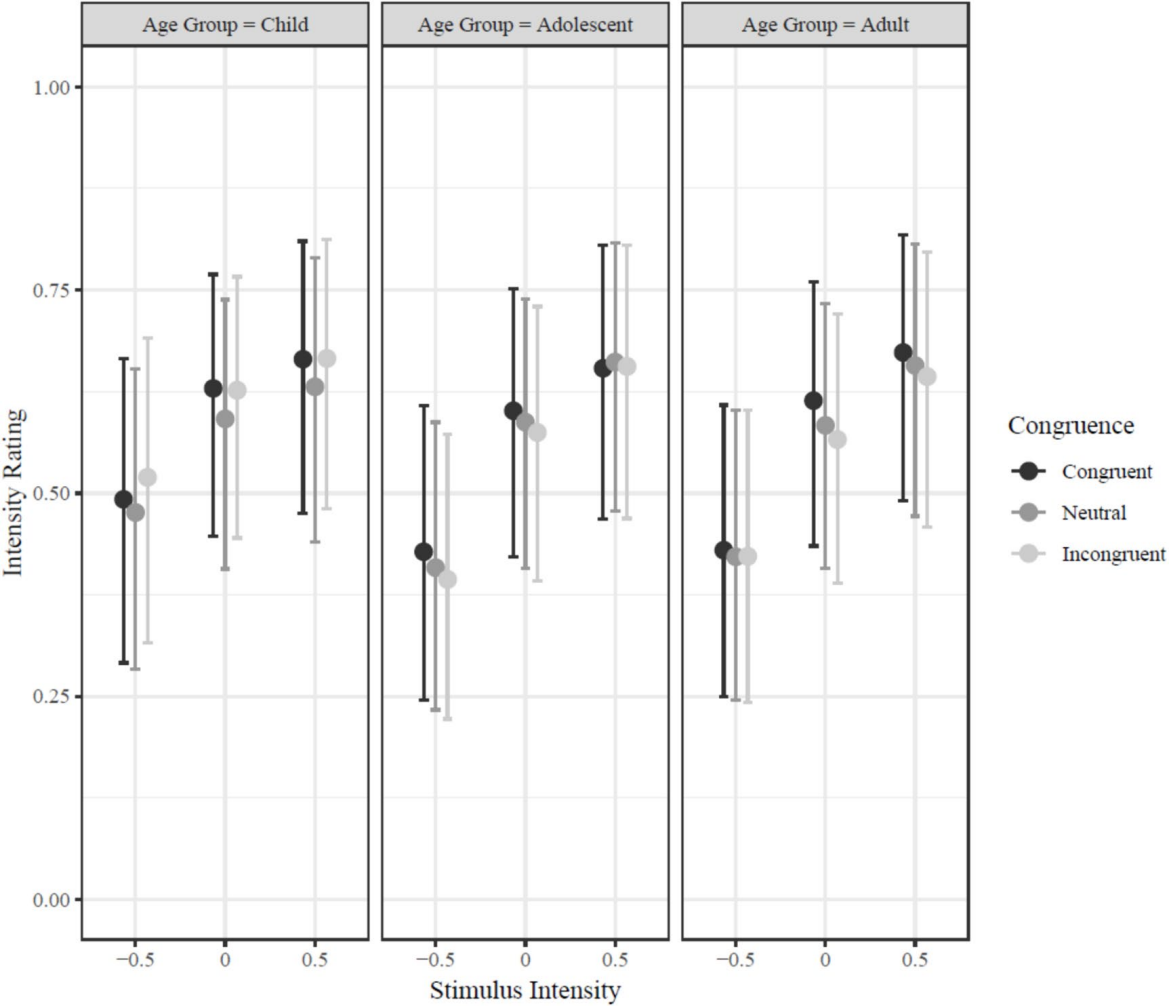
Predicted intensity ratings in Experiment 2 for congruent, neutral, and non-congruent contexts by face intensity (− 0.5 = low, 0 = intermediate, 0.5 = high) for adolescents, adults, and children when no emotion labels were provided. Predicted ratings reflect posterior samples calculated from generalized, multi-level Bayesian regression. Error bars represented 95% credible intervals (meaning that for each interval, there is a 95% probability the true mean intensity rating for the corresponding age group and stimulus intensity level falls within the depicted range). Note that error bars cannot be used to determine reliability because they reflect within- and between-subject variation, whereas reliability of fixed-effect coefficients controlled for between-subject variation

Adolescents rated faces paired with congruent contexts as more intense than those paired with incongruent contexts (md = 0.069; CI_95_ = [0.036, 0.105], *Bayesian-p* < .001). However, children demonstrated the opposite effect, rating faces in congruent contexts as less intense than faces in incongruent contexts (md =  − 0.054; CI_95_ = [− 0.103, − 0.008], *Bayesian-p* = .015). Adults rated faces in congruent and incongruent contexts as comparably intense (md = 0.015; CI_95_ = [− 0.033, 0.062], *Bayesian-p* = .539). As in Experiment 1, the difference in intensity ratings of faces between congruent and incongruent contexts was greater for low-intensity faces than high-intensity faces (md = 0.072; CI_95_ = [0.033, 0.112], *Bayesian-p* < .001). These data are shown in Fig. 4.

#### Hypothesis #2: Younger Children’s Perceived Intensity of Emotions Will Be More Influenced by Context Than Adolescents or Adults, Regardless of the Presence of Emotion Labels

We next examined age-related differences in the impact of contextual congruence on intensity ratings when no emotion labels were provided. As noted above, for both children and adolescents, contextual congruence had a significant effect on intensity ratings. In comparing the magnitude of congruency effects between age groups, children showed a comparable discrepancy in their ratings of congruent and incongruent faces, though in the opposite direction of adolescents (md = 0.124; CI_95_ = [0.073, 0.175], *Bayesian-p* < .001) and adults (md = 0.069; CI_95_ = [0.004, 0.135], *Bayesian-p* = .038). Meanwhile, similar to Experiment 1, adolescents and adults again showed comparable congruency-related differences in intensity ratings (md = 0.054; CI_95_ = [− 0.003, 0.110], *Bayesian-p* = .065).

### Summary of Experiment 2

Consistent with Experiment 1, findings from Experiment 2 indicate developmental differences in how sensitive individuals are to the situational context when evaluating the intensity of others’ emotions. Across both experiments, contextual congruency had a stronger effect on children’s intensity ratings than either adolescents’ or adults’ ratings. The most salient difference in results between Experiment 1 and Experiment 2 was in the direction of this contextual congruency effect among children. When emotion labels were present (Experiment 1), children rated faces paired with congruent contexts as more intense than faces paired with incongruent contexts. Without labels present (Experiment 2), this pattern was reversed. Thus, while situational context consistently influenced children’s judgments of emotion intensity, the ways in which children used this information depended upon the linguistic information available to them.

## General Discussion

Here we report that contextual information influences how people perceive the intensity of others’ emotions. Participants generally interpreted others’ emotions to be of higher intensity when facial movements were congruent with contextual information. Although contextual information influenced the interpretations made by adolescents and adults, context exerted an especially strong influence on the way children perceived emotions. This developmental difference was replicated across two experiments.

Consistent with prior research, the present experiment demonstrated the influence of contextual information on the evaluation of others’ facial configurations (Aviezer et al., 2012; Barrett et al., 2019; Chen & Whitney, 2019; Mesquita, 2022; Martinez, 2019). Extending prior research, the current study indicates that context influences not only categorical interpretations of others’ emotions but also perception of the intensity of others’ emotions.

A potentially important finding that emerged from these data is unexpected and warrants further investigation. Between the two studies, the provision of verbal emotion labels affected how context moderated the perception of emotional intensity among children. When emotion labels were used in Experiment 1, children oriented their judgments around the contextual scenes. When those labels were removed in Experiment 2, the effect was reversed. One possibility is that in the absence of verbal labels, the incongruence between the faces and the scenes led children to conclude that something intense must be occurring to result in the incongruence that they perceived. That is, they perceived the incongruence as something inconsistent with their observations of the world and reasoned that something strongly evocative might be causing it. Another possibility is that verbal labels help children cohere their thinking about or attention to emotions. But when children do not have access to labels, and when the context is at odds with the face, their attention becomes diffused. The current data cannot adjudicate between these possibilities, but future research in this area can test the mechanisms that guide changes in emotion processing early in development.

Most of the existing research in the area of emotion perception has not investigated perceptions of intensity or the developmental emergence of the role of context in emotion perception. The present data suggest that emotion judgments are a developmental process whereby individuals learn to track competing signals and change the way they prioritize information. Early emotion learners have access to a relatively small set of emoters and limited contexts in their environment (Jayaraman et al., 2015). As children age, they gain experience with a greater number of emoters and are exposed to more diverse situations; this increasing variability likely drives emotion learning (Ruba et al., 2022). Consistent with this view, as children age they begin devoting more attention to faces than contextual information when making emotion judgments (Leitzke & Pollak, 2016). Relative to young children, adults appear to readily make interpretations about others’ emotions based on facial configurations (Bar, 2004; de Gelder et al., 2006; Schyns & Oliva, 2010).

### Limitations and Future Directions

The goal of this study was to determine whether and how context influenced judgments about intensity of emotion. In naturally occurring social interactions, changes in both bodily and contextual information are dynamic. Therefore, a next step in understanding how people draw inferences about the relative intensity of other’s emotions might include attempts to measure these processes “in the wild” of social life. For the purposes of this study, we grouped contextual stimuli into emotion categories. But there are myriad ways in which people might interpret or attach meaning to the contexts within which they are interacting in naturalistic settings. We did ask participants to evaluate the contextual stimuli used in these experiments, and each context was evaluated similarly by most participants. But people also indicated that multiple emotion labels could conceivably be associated with each of these scenes. Therefore, a next step in this program of research will be to delve more deeply into the various ways that people might attach meaning to these scenes. Post hoc analyses suggested additional nuances that can be explored with planned analyses that have the power afforded by larger sample sizes.

### Conclusion

Real-world judgments about what we think other people might be feeling involve inferences about the scale or relative intensity of emotions. To successfully navigate the social world, individuals’ behavior choices are fine-tuned to the degree of emotion we believe others are experiencing. We are likely to respond differently to someone who seems displeased versus someone who we believe is enraged, and to someone we infer is disappointed versus devastated. Much has been written about the important role of context in the perception of emotion, and this appears to be true of dimensional judgments of intensity as well as categorical judgments. Making inferences about another person’s internal states is a complex learning task given the high variability within and across individuals and contexts. These data suggest that how people attend to perceptual information changes across development.

## Supplementary Information

Below is the link to the electronic supplementary material.

Supplementary file1 (DOCX 27 KB)

## Footnotes

1 Development of the Interdisciplinary Affective Science Laboratory (IASLab) Face Set was supported by the National Institutes of Health Director’s Pioneer Award (DP1OD003312) to Lisa Feldman Barrett. More information is available online at www.affective-science.org

## Additional Information

### Data Availability

The data and analysis scripts are available at: https://osf.io/nfp7x/?view_only=5e2787eee75b465faf958511eb74f382.

## References

[CR1] Adams, R. B., Jr., Hess, U., & Kleck, R. E. (2015). The intersection of gender-related facial appearance and facial displays of emotion. *Emotion Review,**7*, 5–13. 10.1177/1754073914544407

[CR2] Aviezer, H., Trope, Y., & Todorov, A. (2012). Body cues, not facial expressions, discriminate between intense positive and negative emotions. *Science,**338*(6111), 1225–1229. 10.1126/science.1224313 23197536 10.1126/science.1224313

[CR3] Bar, M. (2004). Visual objects in context. *Nature Reviews Neuroscience,**5*, 617–629. 10.1038/nrn1476 15263892 10.1038/nrn1476

[CR4] Barrett, L. F., Adolphs, R., Marsella, S., Martinez, A. M., & Pollak, S. D. (2019). Emotional expressions reconsidered: Challenges to inferring emotion from human facial movements. *Psychological Science in the Public Interest,**20*(1), 1–68. 10.1177/1529100619832930 31313636 10.1177/1529100619832930 PMC6640856

[CR5] Bürkner, P. C. (2017). brms: An R package for Bayesian multilevel models using Stan. *Journal of Statistical Software, 80*(1), 1–28. 10.18637/jss.v080.i01

[CR6] Bürkner, P. C., & Charpentier, E. (2020). Modelling monotonic effects of ordinal predictors in Bayesian regression models. *British Journal of Mathematical and Statistical Psychology*. 10.1111/bmsp.12195 31943157 10.1111/bmsp.12195

[CR7] Buhrmester, M., Kwang, T., & Gosling, S. D. (2011). Amazon’s Mechanical Turk: A new source of inexpensive, yet high-quality, data? *Perspectives on Psychological Science,**6*(1), 3–5. 10.1177/1745691610393980 26162106 10.1177/1745691610393980

[CR8] Calder, A. J., Young, A. W., Keane, J., & Dean, M. (2000). Configural information in facial expression perception. *Journal of Experimental Psychology: Human Perception and Performance,**26*(2), 527. 10.1037/0096-1523.26.2.527 10811161 10.1037//0096-1523.26.2.527

[CR9] Calvo, M. G., & Nummenmaa, L. (2016). Perceptual and affective mechanisms in facial expression recognition: An integrative review. *Cognition and Emotion,**30*(6), 1081–1106. 10.1080/02699931.2015.1049124 26212348 10.1080/02699931.2015.1049124

[CR10] Chen, Z., & Whitney, D. (2019). Tracking the affective state of unseen persons. *Proceedings of the National Academy of Sciences, 201812250*. 10.1073/pnas.1812250116 10.1073/pnas.1812250116 PMC6462097 30814221

[CR11] de Gelder, B., Meeren, H. K., Righart, R., Van den Stock, J., Van de Riet, W. A., & Tamietto, M. (2006). Beyond the face: Exploring rapid influences of context on face processing. *Progress in Brain Research, 155*, 37–48. 10.1016/S0079-6123(06)55003-4 17027378

[CR12] Elfenbein, H. A., & Ambady, N. (2002). On the universality and cultural specificity of emotion recognition: A meta-analysis. *Psychological Bulletin,**128*(2), 203–235. 10.1037/0033-2909.128.2.203 11931516 10.1037/0033-2909.128.2.203

[CR13] Gao, X. Q. & Maurer, D. (2009). Influence of intensity on children’s sensitivity to happy, sad, and fearful facial expressions. *Journal of Experimental Child Psychology, 102*(4), 503–521. 10.1016/j.jecp.2008.11.002 10.1016/j.jecp.2008.11.002 19124135

[CR14] Green, P. & MacLeod, C. J. (2016). Simr: An R package for power analysis of generalised linear mixed models by simulation. *Methods in Ecology and Evolution*, *7*(4), 493–498. 10.1111/2041-210X.12504. https://CRAN.R-project.org/package=simr.

[CR15] Hess, U., Blairy, S., & Kleck, R. E. (1997). The intensity of emotional facial expressions and decoding accuracy. *Journal of Nonverbal Behavior,**21*(4), 241–257. 10.1023/A:1024952730333

[CR16] Jayaraman, S., Fausey, C. M., & Smith, L. B. (2015). The faces in infant-perspective scenes change over the first year of life. *PLoS ONE,**10*(5), e0123780. 10.1371/journal.pone.0123780 26016988 10.1371/journal.pone.0123780 PMC4445910

[CR17] Kruschke, J. K. (2013). Bayesian estimation supersedes the t test. *Journal of Experimental Psychology: General,**142*(2), 573. 10.1037/a0029146 22774788 10.1037/a0029146

[CR18] Leitzke, B. T., & Pollak, S. D. (2016). Developmental changes in the primacy of facial cues for emotion recognition. *Developmental Psychology,**52*(4), 572. 10.1037/a0040067 26784383 10.1037/a0040067 PMC4824404

[CR19] Martinez, A. M. (2017). Computational models of face perception. *Current Directions in Psychological Science,**26*(3), 263–269. 10.1177/0963721417698535 29307959 10.1177/0963721417698535 PMC5754021

[CR20] Martinez, A. M. (2019). Context may reveal how you feel. *Proceedings of the National Academy of Sciences, 201902661.*10.1073/pnas.1902661116 10.1073/pnas.1902661116 PMC6462075 30898883

[CR21] Mesquita, B. (2022). *Between us: How cultures create emotions*. WW Norton & Company.

[CR22] Mondloch, C. J. (2012). Sad or fearful? The influence of body posture on adults’ and children’s perception of facial displays of emotion. *Journal of Experimental Child Psychology,**111*(2), 180–196. 10.1016/j.jecp.2011.08.003 21939983 10.1016/j.jecp.2011.08.003

[CR23] Noh, S. R., & Isaacowitz, D. M. (2013). Emotional faces in context: Age differences in recognition accuracy and scanning patterns. *Emotion,**13*(2), 238. 10.1037/a0030234 23163713 10.1037/a0030234 PMC4119600

[CR24] Pollak, S. D., Messner, M., Kistler, D. J., & Cohn, J. F. (2009). Development of perceptual expertise in emotion recognition. *Cognition,**110*(2), 242–247. 10.1016/j.cognition.2008.10.010 19059585 10.1016/j.cognition.2008.10.010 PMC2673797

[CR25] Psychology Software Tools, Inc. [E-Prime 3.0]. (2016). Retrieved from http://www.pstnet.com

[CR26] R Core Team (2018). R: A language and environment for statistical computing. R Foundation for Statistical Computing, Vienna, Austria. URL https://www.R-project.org/

[CR27] Rajhans, P., Jessen, S., Missana, M., & Grossmann, T. (2016). Putting the face in context: Body expressions impact facial emotion processing in human infants. *Developmental Cognitive Neuroscience,**19*, 115–121. 10.1016/j.dcn.2016.01.004 26974742 10.1016/j.dcn.2016.01.004 PMC6988095

[CR28] Reschke, P. J., & Walle, E. A. (2021). The unique and interactive effects of faces, postures, and scenes on emotion categorization. *Affective Science,**2*, 468–483. 36046211 10.1007/s42761-021-00061-x PMC9382938

[CR29] Ruba, A. L., & Pollak, S. D. (2020). The development of emotion reasoning in infancy and early childhood. *Annual Review of Developmental Psychology,**2*, 503–531.

[CR30] Ruba, A. L., Pollak, S. D., & Saffran, J. R. (2022). Acquiring complex communicative systems: Statistical learning of language and emotion. *Topics in Cognitive Science,**14*(3), 432–450. 35398974 10.1111/tops.12612 PMC9465951

[CR31] Sauter, D. A., Eisner, F., Ekman, P., & Scott, S. K. (2010). Cross-cultural recognition of basic emotions through nonverbal emotional vocalizations. *Proceedings of the National Academy of Sciences,**107*(6), 2408–2412. 10.1073/pnas.0908239106 10.1073/pnas.0908239106 PMC2823868 20133790

[CR32] Schyns, P. G., & Oliva, A. (2010). From blobs to boundary edges: Evidence for time- and spatial-scale-dependent scene recognition. *Psychological Science,**5*, 195–200. 10.1111/j.1467-9280.1994.tb00500.x

[CR33] Scott, K., & Schulz, L. (2017). Lookit (Part 1): A new online platform for developmental research. *Open Mind,**1*(1), 4–14. 10.1162/OPMI_a_00002

[CR34] Srinivasan, R., & Martinez, A. M. (2018). Cross-cultural and cultural-specific production and perception of facial Expressions of emotion in the wild. *IEEE Transactions on Affective Computing,**14*, 1–12. 10.1109/TAFFC.2018.2887267

[CR35] Tiddeman, B. P., Stirrat, M. R., & Perrett, D. I. (2005). Towards realism in facial image transformation: Results of a wavelet MRF method. *Computer Graphics Forum,**24*(3), 449–456. 10.1111/j.1467-8659.2005.00870.x

[CR36] Van den Stock, J., & de Gelder, B. (2012). Emotional information in body and background hampers recognition memory for faces. *Neurobiology of Learning and Memory,**97*(3), 321–325. 10.1016/j.nlm.2012.01.007 22406473 10.1016/j.nlm.2012.01.007

[CR37] Whelan, R. (2008). Effective analysis of reaction time data. *Psychological Record.,**58*(3), 475–482. 10.1007/BF03395630

