# Supplementary material for: Children’s and Adolescent’s Use of Context in Judgments of Emotion Intensity
Source: Affect Sci. 2024 Sep 26;6(1):117–27. doi: 10.1007/s42761-024-00279-5 (PMC11904079; doi:10.1007/s42761-024-00279-5)
Supplement: Supplementary file 1 — Supplementary file1 (DOCX 27 KB) [file 42761_2024_279_MOESM1_ESM.docx]

# Experiment 2 Free Response Task

At the end of Experiment 2, participants completed a free labeling task identifying the discrete emotions they perceived in the experimental stimuli.

## Procedure

After participants in Experiment 2 completed the main emotion judgment task, they provided free response labels for a randomized and counterbalanced subset of 40 images from the full stimuli set. The free response labeling task was always completed after the emotion judgment task to reduce priming effects. Participants verbally responded to the question “What is this person feeling right now?” In order to standardize procedure across age groups, a research assistant typed the response to each item. Participants were instructed to provide only one label per image.

## Response Coding

To summarize this free response data according to the emotion categories participants perceived, we employed a thesaurus-based coding scheme modeled on one used by Betz, Hoemann, & Barrett (2019). Two independent coders looked up each participant-provided label in the Merriam-Webster online thesaurus and searched the entry for a series of emotion category labels of interest, corresponding to the labels used for stimuli during Experiment 1: angry, disgusted, sad, scared, and happy. Participant-provided labels were coded as agreeing with a given category label if that category label was: 1) an exact match to the participant-provided label; 2) a synonym for the participant-provided label; or 3) a “related word” for the participant-provided label (for example, words “related to” angry include “annoyed” and “seething”). Considering related words ensured that participant-provided labels with more nuance than the category labels were still coded as in agreement with a given category label if the two labels were in the same broad family.

## Results

We grouped free response results by stimulus type – i.e., according to the stimulus’s combination of face emotion, context emotion, and face intensity. In Supplemental Table 1, we report the free response labels most frequently provided for each type of stimulus. In Supplemental Table 2, we report the percentage of responses for each type of stimulus that matched our emotion category labels of interest.

Supplemental Table 1

*Frequent Free Responses by Stimulus Type*

| Stimulus type  (face/context & intensity) | Responses (frequency, in %) |
| --- | --- |
| Anger/anger |  |
| High | angry (17), mad (14), annoyed (14), confused (9), upset (6) |
| Intermediate | annoyed (18), mad (17), angry (14), sad (8), confused (8) |
| Low | mad (16), angry (11), annoyed (11), sad (8) |
| Anger/neutral |  |
| High | confused (16), mad (11), angry (11), suspicious (8) |
| Intermediate | mad (16), angry (14), confused (14), upset (6), sad (5) |
| Low | angry (13), mad (10), bored (9), sad (8), neutral (5) |
| Anger/fear |  |
| High | disgusted (24), mad (11), confused (11), angry (5) |
| Intermediate | disgusted (27), mad (11), angry (11), annoyed (10) |
| Low | disgusted (24), mad (12), neutral (6) |
| Disgust/disgust |  |
| High | disgusted (70), gross (7), angry (6) |
| Intermediate | disgusted (66), gross (7), mad (6) |
| Low | disgusted (41), annoyed (6) |
| Disgust/neutral |  |
| High | disgusted (37), mad (17), angry (14), annoyed (7) |
| Intermediate | disgusted (36), mad (13), angry (11), annoyed (8) |
| Low | annoyed (15), disgusted (11), confused (6) |
| Disgust/anger |  |
| High | disgusted (22), mad (20), angry (18), annoyed (13), upset (6) |
| Intermediate | disgusted (21), mad (19), angry (16), annoyed (14) |
| Low | annoyed (16), mad (16), angry (8), disgusted (5) |
| Sadness/sadness |  |
| High | sad (64) |
| Intermediate | sad (59), scared (8) |
| Low | sad (32) |
| Sadness/neutral |  |
| High | sad (57), upset (5), worried (5) |
| Intermediate | sad (38), scared (11), worried (8), confused (5) |
| Low | sad (9), worried (6), bored (6), confused (6), normal (5), nothing (5), neutral (5) |
| Sadness/fear |  |
| High | scared (41), sad (40) |
| Intermediate | scared (40), sad (20), worried (7), nervous (5) |
| Low | scared (28), sad (9), bored (6), nervous (6) |
| Fear/fear |  |
| High | scared (58), nervous (9), worried (8) |
| Intermediate | scared (59), nervous (9), worried (7) |
| Low | scared (33), nervous (8), worried (7) |
| Fear/neutral |  |
| High | scared (23), sad (15), worried (11), nervous (8), confused (7) |
| Intermediate | scared (21), sad (10), worried (9), nervous (8), surprised (5) |
| Low | sad (11), confused (8), bored (6), nothing (5), neutral (5) |
| Fear/sadness |  |
| High | sad (24), scared (16), surprised (8), worried (7), nervous (5) |
| Intermediate | sad (28), scared (15), worried (8) |
| Low | sad (23), worried (8), indifferent (5) |
| Happiness/happiness |  |
| High | happy (78) |
| Low | happy (19), bored (7), neutral (6) |
| Happiness/neutral |  |
| High | happy (79) |
| Low | happy (15), neutral (10), normal (6), fine (5), bored (5), indifferent (5) |

*Note.* For each stimulus type, all responses with frequency ≥ 5% are reported.

Supplemental Table 2

*Free Response-Emotion Category Match Rates by Stimulus Type*

| Stimulus type  (face/context & intensity) | Free label-category match rate (%) | | | | |
| --- | --- | --- | --- | --- | --- |
|  | Angry | Disgusted | Sad | Scared | Happy |
| Anger/anger |  |  |  |  |  |
| High | **49** | 2 | 12 | 5 | 2 |
| Intermediate | **54** | 1 | 17 | 4 | 0 |
| Low | **41** | 5 | 16 | 6 | 2 |
| Anger/neutral |  |  |  |  |  |
| High | **29** | 4 | 8 | 2 | 2 |
| Intermediate | **38** | 4 | 11 | 2 | 4 |
| Low | **28** | 11 | 12 | 4 | 3 |
| Anger/fear |  |  |  |  |  |
| High | **45** | 26 | 5 | 2 | 1 |
| Intermediate | **61** | 28 | 4 | 2 | 0 |
| Low | **48** | 28 | 8 | 3 | 2 |
| Disgust/disgust |  |  |  |  |  |
| High | 83 | **76** | 1 | 1 | 0 |
| Intermediate | 79 | **70** | 4 | 2 | 0 |
| Low | 51 | **44** | 4 | 2 | 2 |
| Disgust/neutral |  |  |  |  |  |
| High | 77 | **39** | 3 | 2 | 1 |
| Intermediate | 69 | **37** | 5 | 3 | 2 |
| Low | 34 | **16** | 7 | 4 | 5 |
| Disgust/anger |  |  |  |  |  |
| High | 77 | **24** | 7 | 4 | 0 |
| Intermediate | 72 | **21** | 8 | 3 | 1 |
| Low | 47 | **7** | 11 | 4 | 2 |
| Sadness/sadness |  |  |  |  |  |
| High | 4 | 1 | **72** | 3 | 3 |
| Intermediate | 4 | 2 | **67** | 10 | 2 |
| Low | 4 | 4 | **41** | 6 | 4 |
| Sadness/neutral |  |  |  |  |  |
| High | 2 | 1 | **62** | 7 | 3 |
| Intermediate | 5 | 1 | **43** | 12 | 4 |
| Low | 6 | 6 | **16** | 5 | 6 |
| Sadness/fear |  |  |  |  |  |
| High | 0 | 0 | **41** | 49 | 0 |
| Intermediate | 1 | 1 | **23** | 45 | 4 |
| Low | 1 | 6 | **12** | 34 | 1 |
| Fear/fear |  |  |  |  |  |
| High | 2 | 2 | 1 | **70** | 3 |
| Intermediate | 1 | 1 | 2 | **67** | 3 |
| Low | 2 | 5 | 5 | **39** | 5 |
| Fear/neutral |  |  |  |  |  |
| High | 5 | 3 | 17 | **30** | 6 |
| Intermediate | 2 | 3 | 13 | **27** | 6 |
| Low | 5 | 7 | 15 | **7** | 5 |
| Fear/sadness |  |  |  |  |  |
| High | 8 | 6 | 28 | **25** | 2 |
| Intermediate | 5 | 5 | 35 | **22** | 5 |
| Low | 5 | 4 | 28 | **8** | 2 |
| Happiness/happiness |  |  |  |  |  |
| High | 0 | 0 | 0 | 0 | **97** |
| Low | 4 | 8 | 5 | 4 | **28** |
| Happiness/neutral |  |  |  |  |  |
| High | 0 | 1 | 0 | 0 | **94** |
| Low | 5 | 7 | 4 | 2 | **21** |

*Note.* Bolded values correspond to the emotion category labels used for the given stimuli in Experiment 1.

Supplemental Table 3

*Results of simulation-based power analyses.*

| *studyName* | *effect* | *actual_coef* | *actual_z* | *power80_coef* | *powered* |
| --- | --- | --- | --- | --- | --- |
| *unlabeled* | *incTriNum* | *0.214* | *2.93239* | *0.22* | *FALSE* |
| *unlabeled* | *intensTriNum* | *1.913* | *6.103319* | *0.74* | *TRUE* |
| *unlabeled* | *AgeGroupchild:incTriNum* | *-0.218* | *-1.96518* | *0.4* | *FALSE* |
| *unlabeled* | *intensTriNum:incTriNum* | *-0.039* | *-0.21745* | *0.52* | *FALSE* |
| *labeled* | *incTriNum* | *0.647* | *7.481134* | *0.24* | *TRUE* |
| *labeled* | *intensTriNum* | *2.505* | *11.46115* | *0.62* | *TRUE* |
| *labeled* | *AgeGroupchild:incTriNum* | *-0.202* | *-1.61221* | *0.35* | *FALSE* |
| *labeled* | *intensTriNum:incTriNum* | *0.323* | *1.499612* | *0.65* | *FALSE* |

*Note. For computational efficiency these simulations used a maximum-likelihood estimation with a model specification analogous to the reported results in the manuscript. However, for direct comparison to the power results, the maximum-likelihood estimates' coefficients are shown here. The power80_coef column indicates the magnitude of the coefficient necessary to achieve 80% power, given the rest of the model structure remains constant (e.g., quantity of data, random-effects estimates). Here we report the model coefficients' powers corresponding to the primary hypotheses and results in the manuscript.*
